# Falls, Depression and Antidepressants in Later Life: A Large Primary Care Appraisal

**DOI:** 10.1371/journal.pone.0002423

**Published:** 2008-06-18

**Authors:** Ngaire Kerse, Leon Flicker, Jon J. Pfaff, Brian Draper, Nicola T. Lautenschlager, Moira Sim, John Snowdon, Osvaldo P. Almeida

**Affiliations:** 1 Department of General Practice and Primary Health Care, Faculty of Medical and Health Sciences, University of Auckland, Auckland, New Zealand; 2 Western Australian Centre for Health and Ageing, University of Western Australia, Perth, Australia; 3 School of Psychiatry, University of New South Wales, Sydney, Australia; 4 Graduate Higher Degree Systems Program, School of Nursing, Midwifery and Postgraduate Medicine, Edith Cowan University, Perth, Australia; 5 Discipline of Psychological Medicine, University of Sydney, Sydney, Australia; James Cook University, Australia

## Abstract

**Background:**

Depression and falls are common and co-exist for older people. Safe management of each of these conditions is important to quality of life.

**Methods:**

A cross-sectional survey was used to examine medication use associated with injurious and non-injurious falls in 21,900 community-dwelling adults, aged 60 years or over from 383 Australian general practices recruited for the DEPS-GP Project. Falls and injury from falls, medication use, depressive symptoms (Primary Health Questionnaire (PHQ-9)), clinical morbidity, suicidal ideation and intent, health status (SF-12 Health Survey), demographic and lifestyle information was reported in a standardised survey.

**Findings:**

Respondents were 71.8 years (sd 7.7) of age and 58.4% were women. 24% 11% and 8% reported falls, fall related injury, and sought medical attention respectively. Antidepressant use (odds ratio, OR: 1.46; 95% confidence interval, 95%CI: 1.25, 1.70), questionable depression (5–14 on PHQ OR: 1.32, 95%CI: 1.13, 1.53) and clinically significant symptoms of depression (15 or more on PHQ OR: 1.70, 95%CI: 1.14, 1.50) were independently associated with multiple falls. SSRI use was associated with the highest risk of multiple falls (OR: 1.66, 95%CI: 1.36, 2.02) amongst all psychotropic medications. Similar associations were observed for injurious falls. Over 60% of those with four accumulated risk factors had multiple falls in the previous year (OR: 3.40, 95%CI: 1.79, 6.45); adjusted for other demographic and health factors.

**Interpretation:**

Antidepressant use (particularly SSRIs) was strongly associated with falls regardless of presence of depressive symptoms. Strategies to prevent falls should become a routine part of the management of older people with depression.

## Introduction

Older people who fall are twice as likely to be depressed compared with those who do not fall[1]. Depression is common in old age[2], is treatable, and outcomes improve with effective antidepressant therapy, and this could lead to a decrease in the morbidity associated with falls. However, antidepressant use can also increase the risk of falls, both for those in the community[3] and in residential care.[4], [5] The use of psychotropic medications increases the risk of falls[3], but it is unclear whether this risk is the same across a group of medications (for example, antidepressants), specific to a certain class of drugs (eg selective serotonin reuptake inhibitors, SSRIs), or whether the effect of the drug is independent of concurrent mental health morbidity (for example, presence and severity of depressive symptoms).

Falls are an important public heath issue for older people, health providers and policy makers. They are associated with increased risk of placement in residential care [6], hospitalisation [6] and mortality.[7] Up to 35% of all older people fall every year, with 68% of fallers sustaining an injury and 24% requiring health services[1]. Fractures occur because of falls in 4% of cases[8], with hip fractures being associated with the highest morbidity and mortality[6]. For those with depression, the morbidity associated with falls compounds disability. The majority of older people fall once factors distinguishing single fallers from multiple fallers are not well understood.

As part of a randomised trial to improve detection and management of depression in older Australians, the DEPS-GP Project, 20,000 primary care patients, aged 60 years or older, reported information about depressive symptoms, medications, falls and health status. The aim of this cross-sectional study was to investigate the risk factors associated with single, multiple and injurious falls, especially related to depression and psychotropic medication use.

## Methods

### Objectives

A cross-sectional survey of community dwelling older primary care patients was used to investigate associations between depressive symptoms, medication use, falls and fall-related injury.

### Participants

Eligible participants were all patients aged 60 years or over attending family physicians enrolled in the DEPS-GP project, a clustered randomised trial designed to test the efficacy of a family medicine based intervention to reduce depression and self-harm behaviour in later life. Recruitment details are reported elsewhere in detail[9]. Australian general practitioners listed on the Australasian Medical Publishing Company Proprietary Limited database were mailed an invitation to participate; Of the 772 who provided written informed consented for participation, 383 (49.6% of those who consented) contributed to the recruitment of patients. Participating general practitioners held practice registers of all patients enrolled with their practice and they mailed a questionnaire to those aged 60 years and over (n = 77,820 potential target population, on average 203 patients per practitioner), with a request that the questionnaire be returned in a reply-paid envelope even if those contacted chose not to complete it. Of these, 22,251 questionnaires (29%) were returned with written informed consent, 9,087 were returned not completed, 2,934 were returned to the sender because the person named on the envelope was not known at the address, and 820 were not posted by the general practitioner (total response rate = 64.9%). A small proportion of older adults who consented were found to be ineligible because they were under 60 years of age (n = 130). A further 221 had incomplete data on basic demographic characteristics (age and gender) and were excluded from the analysis, leaving a sample of 21,900 older people, of whom 21,596 reported information on falls.

### The measures

All variables were ascertained by self-report on a questionnaire posted to participants and returned in a reply-paid envelope. Age, gender, marital status, highest educational level achieved and current living arrangements were ascertained using standard questions.

#### Medication use

Participants listed their prescribed medications on the questionnaire and they were coded into WHO standard classification of medications groups by a trained research assistant.[10] Antidepressants, hypnotics, anxiolytics, antipsychotics, and other CNS acting drugs that included lithium, antiepileptic and anti-parkinsonian medications were coded for this study.

The Patient Health Questionnaire (PHQ-9)[11] was used to assess *depressive symptoms:* score lower than 5, between 5 and 14, and greater than 14 indicate no, questionable and significant depressive symptoms respectively. *Suicidality* was measured with the question ‘*Have you ever thought about or attempted to kill yourself in your lifetime?*’ The seven possible answers ranged from ‘*no*’ to ‘*I attempted to kill myself and really hoped to die*’. Subjects who acknowledged any suicidal thoughts or behaviour during their lifetime were considered “ever suicidal”. A similar question assessed suicidal ideation over the preceding 12 months.

*Physical activity* was rated as Yes or No answer to the question: “As a rule, do you do at least half an hour of moderate or vigorous exercise (such as walking or sport) on five or more days of the week?” This question has been shown to efficiently discriminate between physically active and sedentary older adults.[12]

*Social support* was measured with the Duke Social Support section of the Duke Multidisciplinary Assessment Tool, asking respondents to decide between 5 levels of response “none of the time” to “all of the time” across questions about relationships with family and friends [13]. A score between 8 (lesser) and 40 (greater social support) was obtained from the eight item option. The score was divided into quartiles for analysis.

The body mass index (BMI) was calculated by dividing self-reported weight (in kilograms) by height (in metres) squared. BMI was categorised: underweight (BMI<18), normal weight (18≤BMI<25), overweight (25≤BMI<30) and obese (BMI≥30).

Common morbidities of old age were ascertained by asking participants if they had ever been told by a doctor that they had arthritis, diabetes, hypertension, stroke, heart attack or angina, heart failure, poor circulation to the legs, asthma, emphysema, osteoporosis and cancer. Arthritis and stroke were left as individual explanatory variables; the remainder were summed and recoded into four categories, 0, 1, 2, and 3 or more co-morbidities.

The two questions assessing *ever* and *current smoking* (“Have you ever smoked cigarettes, pipe, cigars?” and “Are you currently smoking?”) were transformed to never smoked, ex-smoker, current smoker. *Alcohol* use was coded as risk drinking if women reported consuming more than 7 standard drinks in a week and men consumed more than 14 drinks in a week.[14]

Health status was assessed using the SF-12[15] summary scores for Mental Health (SF-12 MCS score) and Physical Health (SF-12 PCS score) (range of scores 0 to 100) dichotomised to less than or equal to 40 and greater than 40 based on the mean score of the Australian population (mean 50, standard deviation 10).[16]

Confidence about not falling was ascertained with the question: “How confident are you that you can do all your daily activities without falling?” with three level responses; not at all, quite and completely confident. Anxiety was ascertained using the anxiety subscale of the Hospital Anxiety and Depression Scale (HADS)[17]; a total score lower than 8, 8 to 10, and greater or equal 11 indicating no, questionable or significant anxiety, respectively.

The primary outcome of interest in this study was *falls*, which we assessed with the following questions: “In the past 12 months have you: slipped, tripped or stumbled? (not including falls to the ground);” “Have you had a fall to the ground? (not including stumbles or trips).” Subjects who answered ‘yes’ to the latter were then asked: “How many falls have you had during the past 12 months?”; “Were you injured as a result of any of these falls?”; “Did you need to seek medical attention (eg doctor, hospital)?” The last two questions recorded injury and severity of injury.

### Ethics

Participants gave written informed consent. The Human Research Ethics Committee of the University of Western Australia (number: RA/4/1/1107) and the Royal Australian College of General Practitioners (number: 20040128) approved this study.

### Statistical methods

The statistical package Stata 9.2 was used to analyse the data. Participants were categorised into “fallers” (one or more falls to the ground) and “non fallers”. Fallers who sustained any injury were further subcategorized and compared with all others, as even injury not requiring medical attention may cause decreased function from pain. Descriptive statistics were calculated for the groups of fallers and non-fallers, and those who had sustained an injury from fall. The association between falls and other measures was examined using univariate methods. To establish those risk factors independently related to single, multiple and injurious falls, a logistic regression model, controlling for all other variables in the model and adjusting the standard errors for the effect of clustering (treating family physician) was used. Accumulated risk was investigated by creating variables describing the group with combinations of risk factors, eg older age, female gender, and by testing the new composite risk variable within the multivariable model.

## Results

This sample comprised community dwelling older people, with only 54 people living in residential care facilities. The average age was 71.8 years (sd 7.7) and 58.4% were women. The majority (62.7%) were physically active and 24.0% lived alone (Table 1). Despite 77.5% of participants reporting good, very good or excellent health, 57.0% reported arthritis, 7.3% stroke, and only 20% had no other co-morbidities. The PHQ score showed that 77.7% of participants exhibited no significant symptoms of depression, however 18.2% had at some time in their lives considered suicide, and 10% had thought about or attempted suicide in the last 12 months. The distribution of falls is shown in Table 2 and characteristics of the participants in Table 1.

**Table 1:** Demographic and Clinical Characteristics of Fallers and Non-fallers

		Fall during past 12 months		
Patient characteristics	Overall	Yes	No	p value
		4,974	15,662	
Age, mean (sd), years	71.8 (7.7)	73.4 (8.3)	71.3 (7.4)	<0.001
Female, n (%)	12,074 (58.4)	3,258 (66.5)	8,816 (56.3)	<0.001
Australian born, n (%)	15,317 (74.4)	3,715 (74.9)	11,602 (74.2)	0.312
Married or defacto, n (%)	13,810 (67.0)	2,945 (59.3)	10,865 (69.5)	<0.001
Living alone vs with others, n (%)	4,948 (24.0)	1,406 (28.4)	3,542 (22.7)	<0.001
Physically active, n(%)	12,831 (62.7)	2,829 (57.6)	10,002 (64.3)	<0.001
University qualification, n (%)	3,073 (15.2)	761 (15.7)	2,371 (15.0)	0.284
Social support score, mean (sd)	33.9 (4.8)	33.1 (5.3)	34.2 (4.6)	<0.001
Social support quartiles, n (%)				
Lowest	5,143 (25.7)	1,531 (32.2)	3,612 (23.7)	<0.001
Second	5,063 (25.3)	1,177 (24.8)	3,886 (25.5)	
Third	4,567 (22.9)	1,024 (21.5)	3,543 (23.3)	
Highest	5,198 (26.0)	1,019 (21.4)	4,179 (27.5)	
BMI group, n (%)				
Normal weight	6,992 (37.1)	1,550 (35.0)	5,442 (37.8)	<0.001
Underweight	321 (1.7)	93 (2.1)	228 (1.6)	
Overweight	7,452 (39.6)	1,689 (38.1)	5,763 (40.0)	
Obese	4,057 (21.5)	1,100 (24.8)	2,957 (20.5)	
Use of medications, n (%)				
Antidepressants	2,460 (12.0)	806 (18.9)	1,654 (10.2)	<0.001
Antipsychotics	433 (2.1)	147 (3.4)	286 (1.8)	<0.001
Anxiolitics	894 (4.4)	264 (6.2)	630 (3.9)	<0.001
Hypnotics	1,024 (5.0)	304 (7.1)	720 (4.5)	<0.001
Other CNS drugs	6,590 (32.3)	1,796 (42.0)	4,794 (29.7)	<0.001
Type of antidepressant, n (%)				
Cyclic antidepressants	742 (3.6)	218 (5.1)	524 (3.2)	<0.001
SSRIs	1,233 (6.0)	441 (10.3)	792 (4.9)	<0.001
MAOI	7 (0.03)	1 (0.02)	6 (0.04)	1.000
MAOI-A	69 (0.3)	13 (0.3)	56 (0.3)	0.768
Other antidepressants	450 (2.2)	153 (3.6)	297 (1.8)	<0.001
Arthritis, n (%)	11,763 (57.0)	3,352 (67.4)	8,411 (53.7)	<0.001
Stroke, n (%)	1,508 (7.3)	520 (10.4)	988 (6.3)	<0.001
Number of other morbid disorders, n (%)				
None	4,321 (20.9)	817 (16.4)	3,504 (22.4)	<0.001
1	6,682 (32.4)	1,354 (27.2)	5,328 (34.0)	
2	5,097 (24.7)	1,265 (25.4)	3,832 (24.5)	
3 or more	4,536 (22.0)	1,538 (30.9)	2,998 (19.1)	
Smoking status, n (%)				
Never smoked	10,628 (51.8)	2,546 (51.8)	8,082 (51.9)	0.964
Ever smoked	8,594 (41.9)	2,062 (41.9)	6,532 (41.9)	
Currently smoking	1,280 (6.2)	311 (6.3)	969 (6.2)	
Alcohol use, n (%)				
Abstain	5,033 (24.7)	1,352 (27.6)	3,681 (23.7)	<0.001
Non-risk alcohol use	10,044 (49.2)	2,327 (47.6)	7,717 (49.7)	
High risk alcohol intake	5,326 (26.1)	1,211 (24.8)	4,115 (26.5)	
Self-rated health, n (%)				
Excellent/very good	7,315 (35.7)	1,280 (26.0)	6,035 (38.8)	<0.001
Good	8,575 (41.8)	2,015 (40.9)	6,560 (42.1)	
Fair/poor	4,603 (22.5)	1,632 (33.1)	2,971 (19.1)	
SF12 – PCS score ≤40, n (%)	6,945 (36.5)	2,351 (53.1)	4,594 (31.4)	<0.001
SF12 – MCS score ≤40, n (%)	1,470 (7.7)	580 (13.1)	890 (6.1)	<0.001
Confidence of no falls, n (%)				
not at all	1,267 (6.2)	713 (14.4)	554 (3.6)	<0.001
Quite	7,718 (37.7)	2,415 (48.9)	5,303 (34.1)	
Completely	11,503 (56.1)	1,811 (36.7)	9,692 (62.3)	
Depressive symptoms: PHQ-9 n (%)				
No depression	14,905 (77.7)	2,926 (65.7)	11,979 (81.3)	<0.001
Questionable depression	3,680 (19.2)	1,250 (28.1)	2,430 (16.5)	
Significant depression	602 (3.1)	279 (6.3)	323 (2.2)	
HADS anxiety grouping, n (%)				
No anxiety	16,612 (84.0)	3,640 (77.7)	12,972 (86.0)	<0.001
Questionable anxiety	1,887 (9.5)	584 (12.5)	1,303 (8.6)	
Significant anxiety	1,268 (6.4)	461 (9.8)	807 (5.3)	
Ever suicidal thought/attempt, n (%)	3,704 (18.2)	1,192 (24.5)	2,512 (16.3)	<0.001
12-month suicidal thought/attempt, n (%)	899 (10.9)	346 (14.3)	553 (9.5)	<0.001

the numbers reported in each of the cells represent the exact numbers of fallers and non-fallers for each of the exposures. However, because of missing values, the denominator was not always 4,974 for fallers and 15,662 for non-fallers

BMI = Body Mass Index

PHQ = Patient Health Questionnaire

HADS = Hospital Anxiety and Depression

PCS = physical component summary score of the SF-12

MCS = mental health component summary score of the SF-12

SSRI = Selective Serotonin Reuptake Inhibitor

HADS = Hospital Anxiety and Depression Scale

**Table 2:** Falls and Injury In a Primary Care Sample (N = 21, 900)

Falls variables	Total, N	Yes, N	%
Slipped tripped or stumbled in last 12 months	21,596	7,612	35.2
Fallen to the ground in last 12 months	20,636	4,974	24.1
1 fall		2,352	47.3 (of those fallen)
2 falls		1,346	27.1
3 falls		694	13.9
4 falls		287	5.8
5 or more falls		295	5.9
Injury from fall		2,351	45.4% of fallers 11.4% of all
Medical attention for injury		1,813	36.4% of fallers 8.8% of all

Number are reported where there was complete data

Falls were reported by 24.1% of participants and 35.2% had tripped or stumbled. Less than half (47.3%) of those who had fallen, fell only once, 27.1% fell twice and the remainder fell 3 or more times. Almost half of fallers (45.4%) reported some form of injury from a fall and 36.4% of fallers (8.8% of the whole sample) sought medical attention for an injury (Table 2). Almost half, 1,080 (46%) of those who fell only once reported an injury and 1,208 (46%) of multiple fallers reported an injury. Twelve percent of the sample were taking antidepressant medication, 2% were taking antipsychotics and 5% hypnotics. Of the antidepressants, SSRIs were the most commonly prescribed (6%), closely followed by cyclic antidepressants (3.6%).

There were several differences between fallers and non-fallers (Table 1), as well as between those who had or had not sustained an injurious fall (Table 3).

**Table 3:** Characteristics of Older People Sustaining Fall Related Injury in a Primary Care Sample.

	Injurious fall during past 12 months		
Patient characteristics	Yes	No*	p value
	2,351	19,549	
Age, mean (sd), years	73,6 (8.5)	71.7 (7.6)	<0.001
Female, n (%)	1,674 (71.3)	11,201 (57.3)	<0.001
Australian born, n (%)	610 (26.0)	5,003 (25.6)	0.693
Married or defacto, n (%)	1,292 (55.1)	13,275 (68.0)	<0.001
Lives alone vs with others, n (%)	707 (30.2)	4,618 (23.7)	<0.001
Physically active, n(%)	1,330 (57.3)	12,324 (63.6)	<0.001
University qualification, n (%)	343 (14.9)	2,832 (14.8)	0.879
Social support score, mean (sd)	33.0 (5.4)	34.0 (4.8)	<0.001
Social support quartiles, n (%)			
Lowest	748 (33.3)	4,680 (24.8)	<0.001
Second	539 (24.0)	4,775 (25.3)	
Third	465 (20.7)	4,376 (23.2)	
Highest	492 (21.9)	5,062 (26.8)	
Body Mass Index group, n (%)			
Normal weight	745 (33.8)	6,655 (37.3)	<0.001
Underweight	49 (2.3)	292 (1.64)	
Overweight	764 (36.7)	7,116 (39.9)	
Obese	525 (25.2)	3,758 (21.1)	
Use of medications, n (%)			
Antidepressants	466 (19.6)	2,200 (11.2)	<0.001
Antipsychotics	80 (3.4)	394 (2.0)	<0.001
Anxiolitics	150 (6.3)	807 (4.1)	<0.001
Hypnotics	188 (7.9)	938 (4.7)	<0.001
Other CNS drugs	1,032 (43.5)	6,094 (31.0)	<0.001
Type of antidepressant, n (%)			
Cyclic antidepressants	121 (5.1)	682 (3.5)	<0.001
SSRIs	261 (11.0)	1,075 (5.5)	<0.001
MAOI	1 (0.04)	7 (0.04)	0.598
MAOI-A	9 (0.4)	67 (0.3)	0.711
Other antidepressants	88 (3.7)	403 (2.0)	<0.001
Arthritis, n (%)	1,642 (69.8)	10,832 (55.4)	<0.001
Stroke, n (%)	264 (11.2)	1,348 (6.9)	<0.001
Number of other morbid disorders, n (%)			
None	338 (14.1)	4,228 (21.6)	<0..001
1	601 (25.6)	6,508 (33.3)	
2	588 (25.0)	4,818 (24.6)	
3 or more	824 (35.0)	3,995 (20.4)	
Smoking status, n (%)			
Never smoked	1,243 (53.4)	10,041 (51.7)	0.288
Ever smoked	938 (40.3)	8,143 (42.0)	
Currently smoking	145 (6.2)	1,219 (6.3)	
Alcohol use, n (%)			
Abstain	674 (29.2)	4,715 (24.4)	<0.001
Non-risk alcohol use	1,064 (46.2)	9,534 (49.4)	
High risk alcohol intake	567 (24.6)	5,064 (26.2)	
Self-rated health, n (%)			
Excellent/very good	559 (24.0)	7,132 (36.8)	<0.001
Good	959 (41.2)	8,145 (42.0)	
Fair/poor	810 (34.8)	4,124 (21.3)	
SF36 – PCS score ≤40, n (%)	1,185 (56.6)	6,203 (34.3)	<0.001
SF36 – MCS score ≤40, n (%)	310 (14.8)	1,266 (7.0)	<0.001
Confidence of no falls, n (%)			
Not at all	376 (16.1)	982 (5.1)	<0.001
Quite	1,184 (50.6)	7,090 (36.6)	
Completely	779 (33.3)	11,304 (58.3)	
Depressive symptoms: PHQ-9 n (%)			
No depression	1,315 (62.7)	14,390 (79.3)	<0.001
Questionable depression	634 (30.2)	3,258 (18.0)	
Significant depression	148 (7.1)	488 (2.7)	
Anxiety: HADS, n (%)			
No anxiety	1,663 (75.4)	15,851 (84.9)	<0.001
Questionable anxiety	297 (13.5)	1,720 (9.2)	
Significant anxiety	245 (11.1)	1,105 (5.9)	
Ever suicidal thought/attempt, n (%)	591 (25.7)	3,311 (17.3)	<0.001
12-month suicidal thought/attempt, n (%)	175 (14.9)	772 (10.2)	<0.001

* Missing data assumed to indicate that no injurious fall occurred during the past 12 months.

PHQ = Patient Health Questionnaire

HADS = Hospital Anxiety and Depression

PCS = physical component summary score of the SF-36

MCS = mental health component summary score of the SF-36

Logistic regression, adjusting for the effect of clustering (family physician), established the independence of the association of depression, activity, antidepressant use with single and multiple falls and fall related injury (Table 4). Analyses were further adjusted for other demographic, health behaviours and health related characteristics. Presence of depressive symptoms and antidepressant use were independently associated with multiple falls and injury but not having sustained a single fall. Those taking as SSRIs were more likely to report a single fall (OR: 1.55 95% CI 1.26, 1.90), sustaining multiple falls (OR 1.66 95% CI 1.36, 2.02) and injurious falls (OR: 1.52, 95%CI: 1.25, 1.84) than those not taking an SSRI (Table 4). The use of cyclic antidepressants, antipsychotic medication, anxiolytics and hypnotics were not associated with falls in this sample.

**Table 4:** The Association Between Risk Factors, 1 Falls, having 2+falls and Injurious Falls in Older Primary Care Patients: Logistic Regression Results (All Factors Forced into the Model*)

		Odds Ratio for 1 falls (95% CI) N = 2,352	Odds ratio for 2+ falls (95% CI)	Odds Ratio for injurious falls (95% CI)
Age group	60–69	1	1	1
	70–79	1.09 (0.97, 1.23)	0.88 (0.77, 1.02)	0.94 (0.83, 1.07)
	80+	**1.41 (1.19, 1.68)**	1.34 (1.12, 1.60)	**1.33 (1.12, 1.58)**
Gender	Female (cw Male)	**1.58 (1.41, 1.76)**	1.11 (0.97, 1.27)	**1.69 (1.49, 1.92)**
Marital status	Married (cw not)	1.07 (0.92, 1.25)	**0.81 (0.70, 0.95)**	**0.84 (0.72, 0.99)**
***Antidepressants***	No	1	1	1
	Yes	**1.34 (1.16, 1.56)**	**1.46 (1.25, 1.70)**	**1.29 (1.12, 1.49)**
Cyclic Adx		1.05 (0.80, 1.39)	1.07 (0.83, 1.39)	0.91 (0.70, 1.17)
SSRIs		**1.55 (1.26, 1.90)**	**1.66 (1.36, 2.02)**	**1.52 (1.25, 1.84)**
MAIOs		1.09 (0.97, 1.22)	1.17 (0.24, 5.61)	0.62 (0.26, 1.49)
Other		1.34 (0.94, 1.91)	**1.54 (1.09, 2.16)**	**1.35 (1.00, 1.83)**
Antipsychotics	Yes	1.06 (0.76, 1.48)	1.33 (0.95, 1.86)	1.07 (0.77, 1.49)
Anxiolytics	Yes	1.04 (0.79, 1.37)	0.90 (0.69, 1.16)	0.94 (0.74, 1.18)
Hypnotics	Yes	1.16 (0.94, 1.43)	1.11 (0.89, 1.38)	1.06 (0.86, 1.32)
Other CNS drugs	Yes	**1.22 (1.09, 1.36)**	1.04 (0.92, 1.17)	1.11 (0.98, 1.25)
Depression	(PHQ-9) None	1	1	1
	Questionable	1.14 (0.98, 1.33)	**1.32 (1.13, 1.53)**	**1.31 (1.14, 1.50)**
	Significant	1.19 (0.86, 1.64)	**1.70 (1.25, 2.31)**	**1.71 (1.27, 2.30)**
Ever suicidal	Yes (cw no)	**1.36 (1.18, 1.56)**	**1.48 (1.29, 1.71)**	**1.37 (1.19, 1.57)**
Anxiety (HADS)	None	1	1	1
	Questionable	0.93 (0.78, 1.12)	1.08 (0.89, 1.30)	1.06 (0.88, 1.26)
	Significant	**0.70 (0.55, 0.89)**	0.92 (0.74, 1.13)	1.00 (0.80, 1.25)
Physical activity	Active (cw not)	1.01 (0.91, 1.13)	**1.21 (1.07, 1.37)**	**1.14 (1.01, 1.29)**
Alcohol use	None	1	1	1
	Non-risk drinking	**1.14 (1.00, 1.30)**	**1.15 (1.01, 1.32)**	1.03 (0.77, 1.38)
	Risk drinking	1.08 (0.93, 1.26)	1.06 (0.90, 1.24)	0.99 (0.70, 1.39)
Arthritis	Yes	**1.25 (1.11, 1.41)**	**1.37 (1.22, 1.55)**	**1.27 (1.13, 1.44)**
Stroke	Yes	1.05 (0.85, 1.29)	**1.23 (1.02, 1.48)**	**1.20 (1.00, 1.44)**
Medical morbidities	None	1	1	1
	1	**0.85 (0.74, 0.98)**	1.14 (0.94, 1.37)	1.02 (0.85, 1.21)
	2	0.96 (0.82, 1.13)	**1.42 (1.18, 1.71)**	1.12 (0.95, 1.33)
	3 or more	1.06 (0.90, 1.25)	**1.15 (1.01, 1.32)**	**1.51 (1.26, 1.82)**
PCS ≤40	Yes (cw no)	**1.16 (1.00, 1.33)**	**1.42 (1.23, 1.63)**	**1.24 (1.07, 1.43)**
Confidence of no falls	Not at all	1	1	1
	Quite	0.93 (0.71, 1.21)	**0.39 (0.32, 0.46)**	**0.67 (0.55, 0.81)**
	Completely	0.79 (0.60, 1.05)	**0.12 (0.10, 0.15)**	**0.40 (0.32, 0.49)**

All analyses were adjusted for the effect of clustering. Living arrangement, social support and Body Mass index were forced into the models but were not independently related to outcomes.

* Antidepressants were initially entered in the model as one group (any antidepressant) and then one at a time (cyclic Adx = antidepressants, SSRIs, MAOIs -including MAOI-A and other antidepressants). cw = compared with, SSRIs: selective serotonin reuptake inhibitors. MAOIs: monoamine oxidase inhibitors. MAOI-A: monoamine oxidase inhibitor A. PHQ-9 = Patient Health Questionnaire. HADS = Hospital Anxiety and Depression. PCS = physical composite score of the SF-12.

Table 5 demonstrates that falls risk accumulated for those with a combination of risk factors, such that women aged 80 years and over with depressive symptoms and taking antidepressants were 1.85 times (95%CI: 1.17, 2.93) more likely to have multiple falls and 2.69 times (95%CI: 1.65, 4.37) more likely to sustain injury. If the antidepressant being taken was an SSRI, 61.6% of the group reported falling and the risk of injury from a fall was 4.96 (95%CI: 2.13, 7.36) compared to those not having that combination of risks.

**Table 5:** Accumulation of Risk for 1 falls, having 2+ falls and Injury from fall according to Age, Gender, Depression and Treatment with Antidepressant Medication

	Prevalence of falls in group n (%)	Odds Ratio for 1 fall (95% CI)	Odds Ratio for 2+ falls (95% CI)	Odds Ratio for injurious falls (95% CI)
Age 80+ years	1,296/3,733 (34.7)	**1.31 (1.14, 1.50)**	**1.44 (1.26, 1.67)**	**1.32 (1.16, 1.52)**
Age 80+ and female	823/2,201 (37.4)	**1.39 (1.16, 1.67)**	**1.35 (1.14, 1.60)**	**1.65 (1.40, 1.94)**
Age 80+ and female and any depressive symptoms*	387/790 (49.0)	**1.43 (1.02, 2.00)**	**1.52 (1.16, 1.97)**	**2.02 (1.56, 2.62)**
Age 80+ and female and any depression* and taking any antidepressant	101/169 (59.8)	1.78 (0.99, 3.22)	**1.85 (1.17, 2.93)**	**2.69 (1.65, 4.37)**
Age 80+ and female and any depression* and taking SSRIs#	53/86 (61.6)	**2.74 (1.29, 5.86)**	**3.40 (1.79, 6.45)**	**3.96 (2.13, 7.36)**

All analyses adjusted for the effect of clustering and controlled for marital status, living arrangements, physical activity, social support grouping, body mass index grouping, arthritis, stroke, multiple medical morbidity, alcohol use, SF-12 physical composite score (PCS), confidence of not falling, anxiety grouping and ever suicide attempt.

* any depressive symptoms = questionable and significant categories combined.

# older adults taking cyclic antidepressants, MAOIs, MAOI-A or other antidepressants were excluded.

SSRI denotes Selective Serotonin Reuptake Inhibitor

## Discussion

A number of factors interacted to multiply the risk of falls, such that over 60% of women older than 80 years with depression and using an SSRI fall and sustain injury. Both depression and the treatment for depression were independently associated to increased risk of falls with SSRI use being associated with the highest risk of falls and injurious falls of all psychotropic agents. Overall one quarter of community-dwelling adults aged 60 years or older reported falling during the preceding 12 months.

We report the risk factors associated with single and multiple falls in a community sample. Distinguishing features between those that report falling once in a year and those that fall more than once have not, to our knowledge, been previously reported. Female gender, usually associated with falls, in this analysis is not associated with sustaining multiple falls, but is independently associated with injury. Depression is independently associated with multiple falls and antidepressants are associated with all falls (1 fall, 2+ falls), and with injury. Of the health issues examined, arthritis and poor health status (as reflected by a low PCS score on the SF-36) were independently associated with all falls and injury whereas stroke was associated with multiple falls and fall with injury. It is possible that differing mechanisms lead to a single ‘careless’ fall compared with multiple falls. Risk factors identified here suggest that there is not necessarily a dose response relationship between depression and falls. Those with questionable and significant depression were more likely to report multiple falls and fall related injury. Antidepressant use however was related to all falls and fall related injury. The true cause of falls may be better revealed by removing the noise of “accidental” falls i.e. people who fall once but have no major risk factors related to the main mechanisms causing falls and thus dilute the relationship. The same proportion who fell once and who fell multiple times reported an injury however, emphasising the need to address all falls in falls prevention programs.

### Depression, antidepressants and falls

Older people currently undertake more then 20% of all primary care consultations while they are 12% of the population [18] and the ageing demographic suggests that their care will make up the majority of primary care within the next half century. Disability associated with depression is greater than that associated with physical conditions and the combination of both physical and mood disorder is associated with the highest levels of disability [19]. Thus appropriate management of both mood disorder and falls is essential to protect quality of life and function for older people.

Independent of other important characteristics leading to falls, those with clinically significant depressive symptoms were more likely to report multiple falls, confirming the findings of others[20]–[23]. Causality cannot be inferred because of the cross-sectional design of the study, but available evidence indicates that depression and depressive symptoms are predictive of falls[24]. Older people with depression have an abnormal gait pattern,[25], [26] and postural abnormalities in the standing position,[27] which suggests a physiological rather than psychological origin for their falls. Depression is also independently related to fractures,[21] dizziness and increased fear of falling.[28] Both fear of falling and depression are independently related to stride-to-stride variability, itself a marker of falls risk.[26]

It is also possible that falls may lead to depression (reverse causality) by reducing functional status and increasing disability[29] ^,^ [30] Whatever the mechanism for the relationship between depression and falls, the presence of one should trigger an inquiry for the other and an offer of appropriate remediation.

In this sample, antidepressants were independently related to an increased risk of falls and injury, confirming studies showing a similar relationship for those in residential care[5] and in the community[31]. One half of those taking antidepressants used SSRIs, and their association with falls risk was higher than for those taking any other psychotropic agents. When SSRIs were introduced, they were not thought to be associated with higher risk of falls, however a recent community cohort study found that SSRIs, but not depression, increased the risk of falls and fractures.[31] It is also possible in the current study that prescription bias occurred, with family physicians choosing to prescribe SSRIs for older adults at increased risk of falls, including those who have depression with dementia. This potential unmeasured confounding could not be adequately controlled for in the current analyses, although health status, physical activity and comorbidities were taken into account.

### Other psychotropic medications

Psychotropic medication use has been recognised as a reversible cause of falls [3], [32]. Hypnotics and antipsychotics were not associated with increased risk of falls in this study, a finding that is in contrast to other research [33], [34]. It is possible that the association between falls and hypnotic/antipsychotic use is stronger in older people in residential care, and that this association is less pronounced amongst independent community dwelling people who do not use these medications as commonly.

### Limitations

Participation in this study was part of recruitment for a randomised trial for family physicians about depression and suicide prevention, and participants were fully informed of the topic. The response rate to this survey is largely unknown. Inaccuracy of practice registers, even in nations that depend on the register for practice payments, can be more than 30%, particularly for the older population [35]–[38] and it has been argued that non respondents from research involving registers should be excluded [39]–[41]. Practice registers are relatively new to Australia and their accuracy has not been established. It is possible that the true response rate to this survey was as high as 60%. The low response rate by family physicians and their patients means that response bias cannot be dismissed however. Response bias will not affect the relationship between risk factor and outcome and the large sample size utilised here made detection of small but important influences on fall risk possible. The risk factors ascertained here were determined by self-report and were not externally validated. Nonetheless, most risk factors identified as independently related to falls in this analysis are biologically plausible and similar to risk factors observed in other studies of older populations [6], [42], [43]. This study was not able to evaluate cognition or balance and gait, which are common risk factors for falls[44]. While these factors limit the interpretation of the results, the information gained here will be of value to primary care providers and researchers interested in the relative risks of depression, and its treatment, on falls and injurious falls in older people.

### Preventing falls

The results of this study have implications for prevention. Cost-effective falls prevention interventions, such as lower limb strengthening and balance retraining[45], and home hazard assessment and modification[46], [47] are now readily available and should be consistently offered to those most at risk, including women over age 80 years with depressive symptoms, especially if a prescription for antidepressants is being written or renewed. In addition, progressive resistance training might be an effective approach to both treat depression and reduce the risk of falls without resorting to antidepressant medication [48].

### Conclusions

Risk factors associated with sustaining a single and sustaining multiple falls differ suggesting potential separate mechanisms for single and multiple falls. Use of antidepressants (most notably SSRIs) and depressive symptoms are independently associated with increased risk of falls in later life. The prevalence of falls with depression means that fall prevention strategies should be a routine part of the management of depression in older people.
